# The complexity of needs and roles of family members during breast cancer rehabilitation: a qualitative study

**DOI:** 10.1186/s12885-024-13200-x

**Published:** 2024-11-21

**Authors:** Marlene Malmström, Annette Holst-Hansson, Ulrika Olsson Möller

**Affiliations:** 1 Department of Health Sciences, Lund University, Lund, Sweden; 2 Institute for Palliative Care, Lund University and Region Skåne, Lund, Sweden; 3 Department of Nursing and Integrated Health Sciences, Kristianstad University, Kristianstad, Sweden

**Keywords:** Breast cancer, Content analysis, Experiences, Family members, Rehabilitation, Typology

## Abstract

**Background:**

Family members play a crucial role in supporting women with breast cancer during their recovery. In the complex situation of being an informal caregiver, their own health and ability to support the patient needs to be acknowledged. The aim was to explore the experiences, needs and roles of family members throughout the rehabilitation process of women with breast cancer.

**Methods:**

A qualitative study was conducted, involving semi-structured individual telephone interviews with 20 purposefully selected family members of women with breast cancer (13 men aged 24–79 years, 7 women aged 19–76 years). Data analysis utilized conventional content analysis and used “casing” as the analysis technique. The study is part of the ReScreen randomized controlled trial and all participants gave informed consent.

**Results:**

The interviews revealed significant variation among family members, leading to the emergence of different typologies based on their reactions and specific preconditions. These typologies included: 1) The case of the assertive and confident team leader, 2) The case of the frustrated but persistent guardian, 3) The case of the reassured bystander, and 4) The case of the neglected outsider. While not mutually exclusive, the cases demonstrated clear similarities and differences in whether individuals felt secure or insecure in the rehabilitation process and their level of involvement in this process. Some described feelings of being involved and active in the process while others experienced not being involved and described feelings of abandonment. However, regardless of their role, family members reported that their own health was seldom considered by healthcare professionals.

**Conclusions:**

This study sheds light on the concept of “we-disease,” where the role of a family member is interrelated with factors such as their health literacy, supporting role, level of involvement, relationship, and identity during the patient's rehabilitation process. This highlights significant divergence in whether family members perceive the rehabilitation process as a collaborative effort or an individual challenge. These perceptions greatly impact their own well-being and ability to support women with breast cancer, underscoring the importance of recognizing family members as informal caregivers and offering tailored support from healthcare professionals when needed.

**Trial registration:**

ClinicalTrials.gov NCT03434717. Registered February 15, 2018.

**Supplementary Information:**

The online version contains supplementary material available at 10.1186/s12885-024-13200-x.

## Background

Estimates of cancer trends point to an increase in cancer incidence of more than 20% by 2040 [1]. This fact will pose an extreme challenge for future cancer care both in terms of use of available resources and ensuring a holistic cancer trajectory that does not merely focus on surviving, but also on rehabilitation and survivorship. Cancer has repeatedly been described as a “we-disease”, [2] as the stress and impact of the disease do not only affect the patient, but all the family members in their everyday life. This means that the increase in cancer incidence will have a corresponding effect on family members, a fact that needs to be considered and addressed in research as well as clinical practice. It has been shown that family members often find themselves in a complex situation where they, in addition to their own fear of the disease and the existential threat it poses, often must support the patient both emotionally and practically throughout the cancer process. This has previously been described as being “co-afflicted” [3]. This concept has similarities with the more commonly used concept of co-dependency, which is underpinned by the fact that severe illness can lead to imbalance and a loss of security for family members [4]. Another aspect that adds to the family member burden is that they also express they need to support the patient in relation to health care. In a study by Jolliffe et al., [5] family members experienced that they needed to supervise health care professionals (HCPs) due to the risk of suboptimal communication and experienced themselves as responsible for bridging communication as well as knowledge gaps. Studies that explore family members’ health point towards negative experiences such as stress-related comorbidities, impairments in health-related quality of life, work productivity, and activity, as well as greater use of health care resources, [6] well in line with studies that showed increased mental and physical illness [7, 8]. Nevertheless, the situation for family members is complex and their experience of supporting the patient varies. In addition to studies that point to an increased risk of stress and morbidity, several studies show that family members have positive experiences in relation to caring for the affected patient, such as gratitude, a sense of accomplishment, and closeness [9–11]. Altogether, this indicates that supporting the patient is a balancing act between burden and meaning. Therefore, to be able to both optimize their own health and enhance their ability to support the patient, family members need to be recognized and acknowledged as caregivers and provided with individualized support. To enable this, healthcare professionals need to have greater knowledge of the experiences and needs of family members and how these can vary between individuals and over time.

Breast cancer (BC) is the most common cancer diagnosis in women worldwide with an annual incidence of approximately 8600 women in Sweden [12]. Developments in BC diagnostics and treatment have gratifyingly resulted in a high survival rate, and in Sweden the 5-year survival rate is 93% [13]. BC has, however, shown to be associated with a wide range of persistent, disabling side effects, such pain, [14] lymphoedema, [15] fatigue, [16] depression, [17] reduced quality of life, [18] fear of recurrence, [19] and distress [20]. With an increasing number of women living with long-term problems, an increasing number of family members will be affected over a longer period of time. Cancer rehabilitation has a health-promoting and person-centered approach where participation of the patient and, if applicable, their family members is crucial for the patient to be supported in managing the situation and experiencing quality of life [21]. Experiences of family members of patients with BC were elucidated by Holst-Hansson et al., [22] showing they were living in a challenging, rearranged, and unknown reality, and that they were struggling to regain ordinary life. They also expressed a need for extended information, support, and guidance from the HCPs to help them endure and cope with the situation.

Patients with BC expressed an ambiguity concerning the changed family roles that occurred, and although they were grateful for the support of family and friends, they struggled with having to depend on them [23]. This became evident in a study of male partners of female BC patients, who experienced that their practical support in running the household and childcare, though necessary, was not always appreciated by their partners [24]. The ambiguity surrounding experiencing the role of a caregiver as positive or strenuous, and the strain a BC diagnosis puts on the family as a unit, family members often express that their need as a family or dyad is seldom seen or met by HCPs [25]. The reasons for the ambiguity and the varied experiences have not been investigated in previous studies, indicating that more research is needed to explore the underlying factors that affect the family members` experiences. Studies suggested that rehabilitation for patients with BC is inadequate in terms of inequality, lack of information regarding rehabilitation, as well as a need for individualization [26, 27], indicating that family members need to support the patient during the entire rehabilitation process. Exploring how family members experience their health, supporting role, involvement, relationships, and identity during the patient’s rehabilitation process will increase knowledge of their prerequisites and needs for individualized support.

## Methods

### Aim

The aim of this study was to explore the experiences, needs and roles of family members of women with breast cancer from time of diagnosis throughout their rehabilitation process.

### Design

An exploratory qualitative method was used, based on conventional content analysis as reported by Hsieh & Shannon, [28] as this was deemed suitable to explore family members experiences of needing, receiving and giving support during the patient’s rehabilitation process.

### Setting

This study focuses on family members of patients with BC. It was conducted as part of the multi-centre research project ReScreen, designed as a randomized controlled trial (RCT). The overall aim of ReScreen is to develop and evaluate a model for an individualised rehabilitation intervention in BC patients. The project was conducted at the BC units at surgical clinics in two hospitals in southern Sweden. The study is described in detail in a study protocol [28]. In addition to exploring the patients’ perspective, ReScreen also focuses on illuminating the perspective of family members. In this study, family members are defined as anyone the patient considers close to them, i.e. not exclusively the individuals from the patient’s family unit.

In Swedish cancer care, patients are often encouraged to have a family member accompany them when visiting the BC unit, but structures for identifying and addressing family members’ needs and concerns are seldom established. During the covid-19 pandemic, when some of the patients whose family members participated in this study were being treated, family members were no longer allowed to accompany or visit patients in Swedish health care due to the increased risk of spreading the infection.

### Participants

From May 2021 to February 2022, patients in ReScreen were, when a questionnaire was distributed, asked in written form whether they had a family member who might be interested in participating in the study. The inclusion criteria were to be able to participate in an individual interview in Swedish, without any limits in age, gender, relationship etcetera. If there was interest, contact details of the family member were enclosed. Sixty-nine family members were approached about the study, and they received mail with information about the study, contact details for the research team, an informed consent form, and a pre-stamped envelope. A total of 35 written informed consent forms were returned between 11 August 2021 and 3 March 2022. Up to two reminders were sent. To ensure variation in relation to age, relationship to the woman with BC, inclusion site, and time, as well as if the patient had received the RCT intervention or not, 26 family members were selected using purposeful sampling. Five of them could not be reached by phone, leaving 21 family members that were informed by telephone about the study (aim, conduct, data security, and confidentiality). Only one family member declined, and the others were scheduled for a telephone interview according to their time preferences. A total of 20 individual interviews were conducted from February to April 2022. For demographic characteristics see Table 1.

**Table 1:** Demographic characteristics of the family members included in the study (*n* = 20)

Variables	
Gender, n	
Male/Female	13/7
Age, mean (SD), range	
Total	52.0 (17.1), 19–79
Men	55.4 (15.5), 24–79
Women	45.7 (18.3), 19–76
Relationship, n	
Husband	4
Cohabitant	8
Mother	1
Daughter	5
Son	1
Brother	1
Patient group, n	
Intervention group	7
Control group	9
Observation group	4

### Data collection

To be able to talk openly about their experiences, while ensuring relevant areas were covered, a semi-structured interview guide (see Additional file 1) with open-ended broad questions and with suggestions of follow-up questions was created by the first and last author during the preparation of the study. Participants were instructed to conduct the interview in an undisturbed location, with no non-participants involved. The telephone interviews were conducted by the last author and started with a presentation of herself and a brief description of the aim of the study and an open-ended question: “What are your thoughts about what you have been through?” The following questions dealt with their perceptions and experiences of their own health during this time, their support functions, factors that facilitated as well as hindered recovery, perceived participation in the process, and their need for support from the health care system (see Additional file 1). After the interview, the interviewer briefly summarized the discussion to ensure the participant's statements were accurately understood. We considered saturation to be reached as various family relationships and several participants from the three groups were included. After the 18th interview, no new information emerged; however, to ensure and confirm data saturation, two additional interviews were conducted.

The interviewer introduced herself as a researcher in the ReScreen project and had no prior relationship with the participants. No one else participated in the interview. The interviews were audio recorded and lasted for 21 to 68 min (median 45.5 min, mean 44 min). Field notes were written by the interviewer after each interview, as a valuable supplement to capture nuances such as participants emotions and tone of voice.

### Data analysis

An inductive approach was adopted since there is lack of knowledge about family members’ experiences and needs in this context and available knowledge is fragmented. Conventional content analysis inspired by Hsieh & Shannon [28] was initially used as a method, as this method is appropriate when research on a phenomenon is limited and therefore requires an inductive approach. The authors (female) have training and expertise in qualitative methodologies and interview studies. The authors come from different professional backgrounds, including surgical and oncological nursing and physiotherapy, ensuring a comprehensive and complementary analytical process. The analysis started with validation of the transcriptions, which consisted of the two authors (AHH, MM) that had not conducted the interviews listening to the interviews and comparing them to the transcripts. Thereafter, the authors independently read transcripts several times to get immersed in the data and obtain a sense of the whole. The initial analysis included coding and was conducted by the authors making headings and margin notes while repeatedly reading the interviews. The codes were then labelled and gathered in a coding scheme and grouped into categories [29]. During this phase it was evident that various “cases” emerged that represented various typologies related to how the family members perceived their situation. Therefore, the continued analysis was inspired by Sandelowski’s description of “casing”, [30] with a back- and forth process of sorting the subcategories and at the same time identify and describe the “cases”. During this process various number and “types of cases” emerged but finally we were able to present four cases in a way that represented the subcategories. Sandelowski described that the casing approach specifies neither any particular methodology nor a specific number of cases. Casing is rather a ‘‘research tactic’’ whereby researchers use cases to manage complexity by an intensive focus on one or more cases purposefully selected for the study. Through this approach, case-oriented analyses help researchers maintain this intensive focus or close connection to those cases [31]. In the presentation of the cases, quotations from participants have been selected for illustration. All names are pseudonyms, and the number represents the interview code.

### Rigor

Efforts were made throughout all the phases to enhance trustworthiness according to the principles of Lincoln & Guba, [32] specifically focusing on credibility, dependability, confirmability, and transferability. To increase credibility, quotations were selected from a variety of family members with different relationships to the patients. Dependability is demonstrated by detailed descriptions of all stages in the analysis process. Credibility and confirmability were strengthened by the involvement in the analysis of all authors (UOM, MM, AHH), who immersed themselves in the data. Under the guidance of UOM, all authors independently conducted the inductive content analysis, followed by auditing and confirmation of category and subcategory relevance through discussion. UOM and MM are experienced researchers within BC rehabilitation, with expertise in physiotherapy (UOM) and nursing in surgical care (MM) and AHH have expertise in oncology nursing and working with women with BC. This diversity enabled researcher triangulation, allowing data to be analysed from multiple perspectives. Differing opinions on data interpretation were resolved through consensus among the authors. All authors possess extensive experience in qualitative research. To further strengthen trustworthiness, the study adhered to the Consolidated criteria for reporting qualitative research (COREQ) guidelines, [33] incorporating a checklist for evaluating various aspects including the research team, study design, and analysis (see Additional file 2). The findings from these elucidating interviews are expected to enrich understanding of the experiences, needs and roles of partners of women with BC throughout the care trajectory.

## Results

**Fig. 1:**
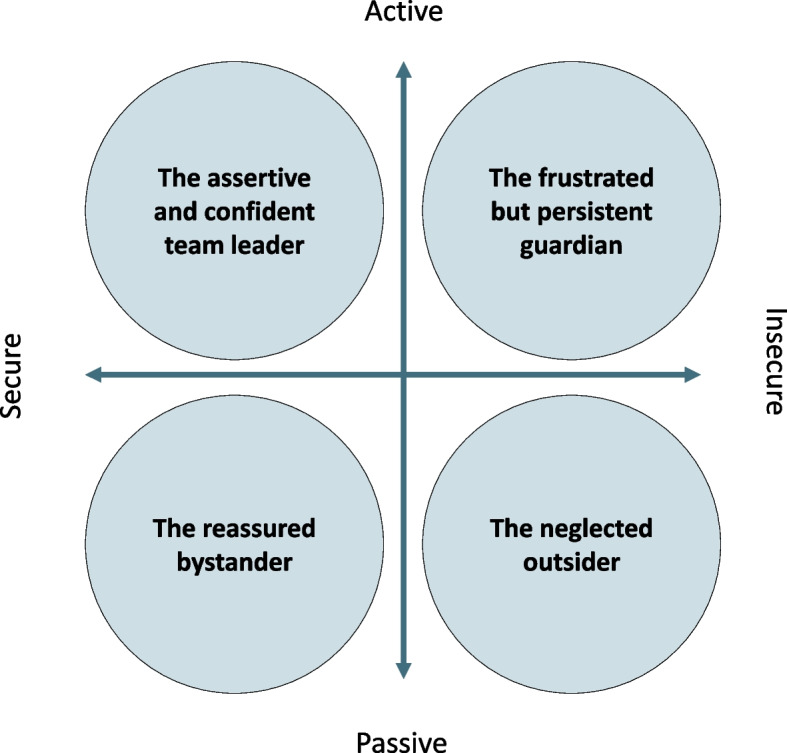
Illustration of the four cases in relation to their role in the cancer process

The analysis revealed considerable variation in the experiences of being a family member of a woman with BC and their experiences were influenced by several factors. A common feature was initial stress and anxiety lasting for different timespans, depending on what occurred during the process and the family members’ previous experiences. Different typologies emerged, which were constructed by how they reacted in this situation, but also depended on the specific preconditions they had. The results of the analysis describe these typologies in the form of four cases: 1. The case of the assertive and confident team leader, 2. The case of the frustrated but persistent guardian, 3. The case of the reassured bystander, and 4. The case of the neglected outsider (Fig. 1). These typologies should be understood in the light of their description and perception when they look back and reflect on the process. This means that the cases should not be viewed as static, but rather as a shifting process where one can go in and out of different cases depending on the situation. Likewise, the cases should not be seen as mutually exclusive, as some have the main characteristics of one case, but also elements of others.

### The case of the assertive and confident team leader

Being an assertive and confident team leader was described as being active and trusting one´s own ability, but also as experiencing trust in medical science and healthcare. Initial concerns at the time of diagnosis were early replaced by rational thoughts of “we can handle this” and a strategy to find adequate information was initiated quickly after the diagnosis. Taking an active part in exploring reliable information sources was experienced as a natural part and an obvious way to act to be able to be a supportive family member. “*One has to stand strong and hold the fort*” as George (*husband, participant 12*) described it.

A core strength of the competent family member was the capacity to retrieve and understand disease-related information from different perspectives and sources. Yussuf, the husband of Anna, had an extended knowledge of medical terminology. He described that he knew how to search for scientific and credible knowledge and found a sense of security in finding adequate and good information, for example in the form of care programs and national guidelines leading to a feeling of being fully updated and involved. As Yussuf said:

*“If you are used to medical terminology, it is easier to absorb the information..//.. and I have searched for a lot of information myself, I know roughly where to look. //.. then it's not that hard to find information about what my wife has suffered” (husband, participant 11).*

Carl, the husband of June, experienced it as self-evident to be supportive and encourage June in the new situation and it was not perceived as problematic or particularly burdensome. Carl made sure to be involved as much as June wanted but took a step forward when June´s needs were not met in terms of e.g., managing finances, but also practical tasks such as giving her injections. Carl had no problem to personally contact health care if needed. Similarly, Michael, the husband of Carol, described that at different occasions he needed to push for Carol´s right to receive e.g. professional counselling when she was emotionally exhausted. Both Carl and Michael described these actions of taking lead when prompted as essential and self-evident.

The family members in this case often had previous experience of healthcare in terms of either being a patient/family member themselves in the past or being employed within healthcare or in clinical research, and therefore knew what to ask for or even demand. Michael used his competence to navigate the system to enable him to join Carol for some of the appointments despite that it was prohibited due to the ongoing pandemic. This way of stretching the rules, or navigating around the rules, stood in contrast to perceptions in other cases where family members didn't even think that this was an option. Michael described it as:

*It was clearly stated that you are not allowed to bring a family member. But she was so emotionally affected that morning [surgery] and she was scared and worried, she has never had any major contact with healthcare and for her this is a little unknown, a little scary. So then I went along, and I was there when the guide wire was placed. And I was there until she went in for surgery …//……so in that we broke a little against these actual rules that existed on a couple of occasions” (husband, participant 15).*

These family members described that they, by having access to the information and knowledge they needed, could handle the situation more easily. By keeping themselves informed about the care process they experienced trust that the HCPs had routines on how the process was to be carried out, what steps were to be taken and what happened in the event of any deviations. The family members in this assertive and confident team leader case experienced genuine trust in the HCPs, especially in specialist care, perceived them as experts and professionals, and that if one just follows what they recommended, one would be fine.

### The case of the frustrated but persistent guardian

Being a frustrated but persistent guardian was characterized by family members who actively participated in the process but were not able to support the patients or solve challenges to the extent they wanted. In this case, insecurity is the key component, in terms of consistently struggling with different practical or emotional concerns which manifested itself as a feeling of not being in control, not being listened to, and not having an overview of the situation. In this case, the family members often described themselves as disappointed or frustrated about the health care system.

For Janet, wife to Cecilia, who was used to solving things, it was frustrating not knowing what to do and not being able to “fix it”. She had a close relationship with Cecilia and the surrounding social network, and she took an active part in the process by being physically present if possible, during hospital visits and by giving emotional and practical support. She sometimes sensed that she had to protect her wife and be her guardian but that it was hard. Another example of taking on the role of being a guardian was described by Holly, the daughter of Ruth. Holly was also frustrated about not having enough information and was disappointed with the contacts with the healthcare system due to lack of communication and insufficient information. When asked whether they got individualized information, Holly said:

*Yes, but not about how she would feel during the treatment, only about the treatment itself, what it is, in general. /…/ and then… Everyone is an individual, so it's very individual how you react, but I think … There should be someone saying… // "you might feel these general things" or "if you're more tired, it's common. You don't need to be worried." (daughter, participant 17).*

In line with Janet and Holy, Jonas felt that he was not sufficiently involved by the health care services in the care of his mother Susan. He experienced the information as fragmented and unclear, which led to him not having an overview of the whole process, and this perceived lack of knowledge led to uncertainty about what would happen next. For Jonas, there was a duality regarding information addressed to his mother and his own needs. He took part in the information they received together but never felt that any information was addressed specifically to him. When asked what kind of support he had and if he knew where to turn to for support, he answered:

*” If I felt that I needed to ask someone, I didn't have a very good grasp of it [were to turn], of course we had her [the contact nurse] number at home, my mother's contact from the breast unit. But it wasn't as if I had been given it, it was she who had been given the contact number, so I didn't know whether I could ask questions or not//..It [the information] was only sent to my mother. And I had to read through hers, it was not really about me." (son, participant 24).*

Holly was disappointed and frustrated about not being involved despite her having unique knowledge about her mother that no one else had. She felt that this knowledge would have been useful to the health services in helping her mother and that she could support her in a way that no one else could. The fact that her knowledge was unrecognized was frustrated and Holly said:

*“.. I can see in a completely different way [than the HCPs], because I have known my mother for 35 years...//..I can see in a different way than someone who has only met her for an hour or so (daughter, participant 17).*

The family members in this case had in common that they were to some extent included in the process but did not receive the support they felt they needed and that they were not involved in the care process to the extent they wanted. They also expressed that they found the information they received to be inadequate, and they did not know where to turn to get help. As they did not know how to handle the situation, they were also unsure of how and when to provide support to the patient, but at the same time perceived that they could support the patient in a way that no one else could.

### The case of the reassured bystander

The reassured bystanders were characterized by feeling confident and secure and being satisfied with the entire care process, without taking on an active role. It was obvious that they felt secure when the patients themselves experienced that they were able to manage and be in control of the situation. In this case, they didn’t need to be in control since they trusted the process and the competence within it. In the reassured bystander case, the family members often took a passive role and were confident that they got all information that they needed. They were also confident that the patient could manage the situation quite well on their own.

Burt, the husband of Atika, was of course worried about his wife’s diagnosis in the beginning but experienced that he was quite quickly reassured by the good reception and information they received from the HCPs. He himself had good and bad experiences with the healthcare system but felt that he was supported well in this situation and that the HCPs were experienced and active, and this made him feel involved and safe. When possible, Burt accompanied Atika to the health care visits, but mostly as a passive bystander for moral support. Sometimes he and Atika had discussed beforehand what questions they had and then they perceived it was unnecessary for him to come along. He trusted Atika to inquire about what they didn't know and tell him afterwards. Burt described the support Atika had received:

*… “she has asked them when she has had medical tests and then learnt more about it…//…She has received very good support from those who have taken care of her, I can say that from what she has told me herself that she has been very satisfied." (husband, participant 30).*

Another aspect of being a reassured bystander was described by Alan, brother of Vera. He genuinely felt that the new situation had affected his life, and he could certainly feel that he was putting his own needs aside, but this was not seen as a problem and felt completely natural. He took note of the information provided by the health service both orally and in writing and was satisfied with it and had confidence in the HCPs. In contrast to the case of the assertive and confident family member, he did not experience that he needed to take control or that he needed to seek additional knowledge.

Another aspect of being a reassured bystander was not expecting any support from the health care system for themselves. Alan even expressed that it was presumptuous to expect any support from the health care system, and that it was his own responsibility to take care of himself.

*”I don't know how much involvement the healthcare system has in caring for family members, because sometimes they can be very busy. But then it's better that you get involved yourself, rather than just waiting for "yes, but shouldn't they call me?". /…/ I don't know if they're [family members] asking too much, or if it's too much for them that they have to help too much. It's up to the individual, actually, I think. You have to take some personal responsibility." (brother, participant 14).*

The family members in this reassured bystander case had no great need to get involved and get support from their social network, and they had agreed with the patient that they wanted to keep it to themselves. To them it was often enough that those closest to them knew about their situation and they stated that it was enough to talk to the patient about what happened and about their concerns and emotions. At the same time, they experienced that if they had a need, they knew that there were people in the immediate vicinity that they could turn to.

### The case of the neglected outsider

Some family members described a feeling of being an outsider in terms of being neglected by either the patient, the social network, or the healthcare system (or all at the same time). Being an outsider raised a feeling of being abandoned and insecure since all focus was on the patient. These experiences were clearly different from those described in the case of the reassured bystander who consciously assumed a passive role and was satisfied with that. On the contrary, the neglected outsider wanted to be involved but for various reasons was not able to.

Mark, who lives with Mary, felt that being an outsider generated a sense of exclusion and uncertainty that in turn led to thoughts about others knowing more than him. He described that his possibility to be involved was based on conditions that were set by Mary who gave him filtered information. When he asked for more information, the answers were often experienced as short and unclear. He also described that he was never invited to attend the medical appointments, which led to experiencing a feeling of loss of control. When Mary was worried and wanted to share emotional and stressful thoughts, he was therefore not in a position to support her in an informed way. This made him feel insecure, he described:

*"I could have given her more feedback if I knew more about what it was about. But I only hear her version. Like, if I had received it from someone else, I might not have interpreted it the same way. /…/ I would have been better off knowing, if I had known more about the disease itself and different stages and so on in order to be able to provide different support, feedback when discussing things." (husband, participant 4).*

He described that it was difficult to know what to ask his wife and when to do it. He was afraid of stepping on her toes and was unsure of the reaction he would get. Therefore, he had to constantly adapt to her, which felt like a difficult balancing act. Similarly, Sam, the daughter of Clare, expressed that not being involved gave her feelings of insecurity and not knowing how to act or support the patient. She said:

*"Well, after all, even if you're like "oh, I'll tell you everything" but you don't, not everything is said, not everything is known. So much also becomes a concern about what is it that I don't know, because I know that there is definitely a side that I don't know and then it becomes more like trying to fish [for information] because you want to be a support." (daughter, participant 3).*

During the interview when Sam retrospectively had time to reflect, she realized that she had not thought about her own needs and that she had pushed her own needs aside during the care process. This affected both her own wellbeing and her prerequisites to support her mother.

The family members in this neglected outsider case clearly felt that they did not have the knowledge and prerequisites to take an active role in the process. This resulted in them not being able to either support the patient or care for themselves by articulating their own needs. To these family members it was evident that they were disappointed with not having been asked about their own well-being or needs, neither when they attended the health care visits, nor through any other information directed to them as family members.

In summary, there were clear similarities and differences between the cases (Fig. 1). The typologies *The case of the assertive and confident team leader* and *The case of the reassured bystander* were largely satisfied with the involvement, support and treatment they received during the process (secure), but differed regarding the need for involvement (active vs passive). However, the typologies *The case of the frustrated but persistent guardia*n and *The case of the neglected outsider* expressed a common dissatisfaction with the support they received and therefore felt insecure. Similarly, difference related to involvement emerged in that the first felt involved (active) while the second did not (passive). However, what the different typologies had in common was that their own health had rarely been recognized by the HCPs during the process.

## Discussion

Aligned with previous research, our study confirms the description of BC as a "we-disease" [2] and highlights family members as being "co-afflicted" [3]. Our results, however, add a more nuanced picture of these concepts by elucidating that the role of a family member is interrelated with factors such as their health literacy, supporting role, level of involvement, relationship, and identity during the patient's rehabilitation process. Our results also show that there is a substantial divergence in whether the family member experience the rehabilitation process as a collaborative effort, corresponding to the “we-disease” concept, or as an individual challenge. This was described through a variety of experiences ranging from the family member being the one who takes all the responsibility (active and secure) to family members feeling excluded and abandoned (passive and insecure), reflecting different ways of relating to the situation and thus having different needs for support. This study therefore provides vital knowledge about the importance of identifying and asking the family members about their needs to optimize the support from health care.

The multitude of experiences categorized into four cases in our study aligns with the study by Joliffe et al., [5] focusing on challenges faced by caregivers. In their study, psychological concerns and emotional strains related to the caregiving role were described, along with the impact on the caregivers' identity. In line with Joliffe et al., [5] and Holst-Hansson et al., [22] our results show that family members often express concerns about not knowing how to best support the person with cancer and how to manage the uncertainty in their situation. Our results also add an important perspective by highlighting a notable variability in the factors influencing family members’ ability to support patients, which seems to depend on their partnership with the healthcare system, social network, and the patient, highlighting the importance of individualized support. In addition, our results show a significant diversity in the support needs of family members, suggesting that those with confidence in their ability to gain and understand relevant information (secure) expressed limited support needs, while other family members articulated feelings of abandonment and expressed disappointment in not being acknowledged as individuals (insecure). This point towards the importance of identifying the individual support needs of family members, both to enhance their experiences and to ensure they have the resources to support the patient effectively, that is to take an active instead of passive role in the patients’ rehabilitation process.

Considering the well-established risks of health issues in family members of people with cancer, including stress-related comorbidities, reduced work productivity, and reduced health-related quality of life, [7] individualized support can yield substantial benefits by, when needed, enhancing family members' health and consequently enhancing their ability to provide sufficient support to the patient. However, research specifically focusing on interventions aiming to enhance the family members’ ability to support the women during their rehabilitation process has previously concerned support during treatment [34] or in terminal care, [35] whilst research about their situation during the patient’s rehabilitation process is lacking. Our results, complemented by insights from a systematic review on nursing interventions [36] for patients with cancer and their family members, underscore the importance of providing support to family members. The review identified three interventions incorporating skills training for family caregivers to manage patients' symptoms, aiming to empower caregivers through information, instructions, quick feedback, and counselling. While the systematic review showed diverse outcomes, with two studies demonstrating improvements in quality of life (QoL), family functioning, and reduced burden for caregivers, one study indicated increased self-efficacy without a corresponding improvement in caregivers' psychological well-being [36]. Altogether, this further emphasizes the critical role of supporting family members, not only for their own well-being and capacity to assist the patient, but also for mitigating the health-economic burden imposed by the disease on society. The insights gained from our study, combined with the systematic review, contribute valuable knowledge that can serve as a foundation for future interventions in this area.

Since previous research has confirmed that women with BC sometimes experience persistent problems with decreased QoL, [37, 38] another important aspect to consider is the longitudinal perspective in terms of family members’ needs throughout the rehabilitation process. This emphasizes the significance of following individuals over time and adapting the support accordingly. Although the participants in this study were not followed longitudinally but rather reflected on the process retrospectively, it was evident that they had undergone a roller coaster experience meeting different challenges throughout the patient’s rehabilitation process, including periods of high stress as well as calm periods where everyday life continued as ordinary.

### Strengths and limitations

This study has strengths and limitations. The study included family members that the patients considered being close to them and could therefore be considered a representative sample. Based on the variability of included participants, the results are transferable to family members to women with BC, and the typologies can represent a wide variety of family members. One of the advantages of 'casing' in this study is that it provides a clear, clinical depiction of how a family member might react in such a situation. However, it is important to point out that this in no way represents family members as a whole but merely aims to illustrate variation, acknowledging that, of course, there are many more variations. The study was based on a robust sample of 20 participants that gave rich and thick descriptions of the subject where data saturation was achieved. The interview guide was developed by UOM and MM and all authors listened to the recorded interviews and validated the transcribed material. Also, the researchers have extensive experience of conducting research in the field of BC rehabilitation and in qualitative research with individual interviewing. A limitation to credibility was that no repeated interviews or member checking to verify the accuracy of the interviews was conducted [31]. Also, to increase variation, other sociodemographic factors such as educational level, or the severity of patients' illness could have been considered as important variables in the purposeful sampling. This would have ensured that different vulnerable groups were represented, which could enhance transferability. Finally, the interviews were conducted directly after the COVID-19 pandemic, where family members’ access to health care were limited. This must be considered when interpreting the results of this study.

## Conclusions

This study illuminates the concept of “we-disease” and demonstrates that family members' experiences divert significantly, influenced by their perception and understanding of how they and the patient perceive their care. Family members may take an active or passive role and feel involved or excluded, showing that for some it is a collaborative effort, while for others it is an individual challenge. This insight emphasizes the interconnectedness of the experiences of both patients and the family members and affects the family members’ well-being as well as ability to give support. It highlights the need for a comprehensive and supportive approach, emphasizing the importance of prioritizing early recognition and systematic screening of family member’s needs. Providing information specifically tailored to them, along with access to dedicated support when needed, emerged as crucial components for experiencing effective support from health care.

## Supplementary Information

Additional file 1. Interview guide.

Additional file 2. Consolidated criteria for reporting qualitative research (COREQ) checklist.

## Data Availability

The data for this study consists of interview transcripts that contain sensitive personal information. Due to restrictions imposed by the Swedish Ethical Review Authority and the consent given by participants, we are unable to share these data publicly. Participants agreed to participate exclusively in the ReScreen study. However, de-identified excerpts are included in the paper. Requests for additional information can be directed to the Swedish Ethical Review Authority at registrator@etikprovning.se.

## Abbreviations

BC: Breast cancer
COREQ: Consolidated criteria for reporting qualitative research
HCPs: Health care professionals
RCT: Randomized controlled trial
