# Supplementary material for: The complexity of needs and roles of family members during breast cancer rehabilitation: a qualitative study
Source: BMC Cancer. 2024 Nov 21;24:1430. doi: 10.1186/s12885-024-13200-x (PMC11580357; doi:10.1186/s12885-024-13200-x)
Supplement: Supplementary file 1 — Additional file 1. Interview guide. [file 12885_2024_13200_MOESM1_ESM.pdf]

Additional file 1. Interview guide

What are your own thoughts about what you've been through?

- What are your thoughts about your own health during this time?
- Could you talk about what you've just told me with anyone? Your (family member)? Someone else?
- Where did you find your strength?
- When did you move from the initial acute crisis/shock phase? What happened? How can this be facilitated? Which factors facilitated or hindered your situation?

How could you support your affected family member?

What are your thoughts about being involved in this situation?

What expectations did you have of healthcare regarding receiving support for yourself?

- Is there a specific period when the need for support from healthcare is greatest?
- What support did you specifically need?
- How can we in healthcare know when and how you need support?
- In the best of worlds, what would the support have looked like?
